# Minimal synthetic inducible promoters for host response to parasitic plants

**DOI:** 10.1093/pcp/pcag101

**Published:** 2026-07-20

**Authors:** Imen Tanniche, Hope Gruszewski, Sukhmanpreet Kaur, James H Westwood

**Affiliations:** School of Plant and Environmental Sciences, Virginia Tech, 401 Latham Hall (0390), Blacksburg, VA 24061, USA; School of Plant and Environmental Sciences, Virginia Tech, 401 Latham Hall (0390), Blacksburg, VA 24061, USA; Department of Biological Sciences, Virginia Tech, 2125 Derring Hall (0406), 926 West Campus Drive, Blacksburg, VA 24061, USA; School of Plant and Environmental Sciences, Virginia Tech, 401 Latham Hall (0390), Blacksburg, VA 24061, USA

**Keywords:** *Arabidopsis thaliana*, *Cuscuta campestris*, minimal synthetic inducible promoter, parasitic plant, *Phelipanche aegyptiaca*, transcription factor binding sites

## Abstract

Parasitic plants are a problem for world agriculture as their direct feeding on hosts can cause high yield losses. One approach to controlling parasitic plants would be to engineer host plants for resistance by expressing an effective defense in response to an invasion by the parasite. While much attention has focused on identifying suitable resistance genes, little work has addressed the challenge of identifying promoters to drive the specific expression of such genes. To meet this need, we set out to develop a parasite-inducible promoter. Starting with previously generated RNAseq data from *Arabidopsis thaliana* parasitized by *Cuscuta campestris*, we identified host genes induced by the parasite and then studied their regulatory regions to identify *cis*-regulatory elements. These elements, or transcription factor binding sites (TFBSs), were recombined into a set of minimal synthetic inducible promoters (MSIPs), fused to the β-glucuronidase (GUS) reporter gene, and stably transformed into *A. thaliana*. The MSIP lines were evaluated with and without *Cuscuta*, and GUS staining showed that most of the MSIPs were induced by *Cuscuta* parasitism, although they were also expressed in some non-parasitized tissues. The most parasite-specific MSIP contained stress-inducible TFBSs, and was also responsive to wounding, crushing and jasmonate treatment. Furthermore, *A. thaliana* parasitized by the root parasite *Phelipanche aegyptiaca* showed similar MSIP induction patterns in the roots, suggesting that at least two types of parasitic plants trigger the same host transcriptional machinery. These MSIPs provide insights into host–parasite interactions and are potentially valuable for driving the expression of novel parasite resistance genes.

## Graphical Abstract

**Graphical Abstract:**
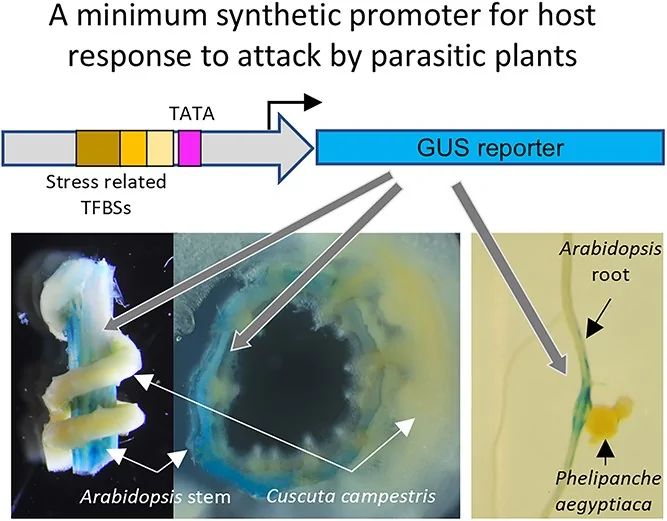

## Introduction

Field dodder (*Cuscuta campestris*) is an obligate parasitic plant that relies on a host plant to survive and reproduce. *Cuscuta* species connect to their hosts by coiling around host stems and producing specialized organs called haustoria, which grow invasively into the host and fuse with its vascular system to obtain nutrients and water (Furuhashi et al. 2011, Olsen et al. 2015, Shimizu and Aoki 2019). Because they feed directly on host resources, parasitic plants can be exceptionally destructive when they attack crops, and *Cuscuta* species, along with members of the root-parasitic family Orobanchaceae, are among the world’s most damaging weeds. For example, *Striga* spp. (witchweeds) are a substantial constraint on cereal production in sub-Saharan Africa (Scholes and Press 2008, Spallek et al. 2013). The broomrapes (*Orobanche* and *Phelipanche* spp*.*) impact legume, vegetable, and oilseed crops across the Mediterranean and Middle East regions (Parker 2012). *Cuscuta* spp. have broad host ranges and a global distribution, thereby impacting a wide range of broadleaf crops (Costea and Tardif 2006, Masanga et al. 2021).

Parasitic weeds are challenging to control by physical and chemical strategies because their close association with the host crop limits access to the parasite without the risk of collateral injury to the crop (Eizenberg et al. 2013). *Cuscuta* spp. have a leafless vining growth habit that tightly coils around host shoots, leaving scant targets for sprays and hampering physical removal. The root parasites of the genera *Orobanche, Phelipanche*, and *Striga* are even more challenging, as their connections to the host occur underground, out of the sight and reach of growers. The physiological integration of parasite and host also complicates chemical control measures because few herbicides are capable of selectively killing a parasite without inflicting damage on the crop host. An ideal method for controlling parasitic plants would be the generation of hosts that are resistant to parasitism. Unfortunately, the identification of resistance traits and breeding for such resistance have been slow, in large part because most affected crop species lack insufficient resistant germplasm to use in breeding programs (Cuccurullo et al. 2022). The use of genetically engineered resistance mechanisms has been proposed as one approach to overcome this constraint (Fernández-Aparicio et al. 2020, Aly et al. 2021).

Genetically engineering a host plant to resist parasitism requires two main elements: (i) an effective defense response and (ii) the regulatory machinery to appropriately express the defense mechanism. Regarding the effective defense component, few cases of host resistance have been well characterized, with most of these exhibiting polygenic control (Albert et al. 2020). For *Cuscuta*, just two examples of resistance have been elucidated. One is specific to tomato (*Solanum lycopersicum*) resistance to *Cuscuta reflexa* and involves the tomato leucine-rich-repeat receptor protein, designated CuRe1, which recognizes a *C. reflexa* glycine-rich protein, termed CuGRP, in the parasite cell wall and induces a hypersensitive reaction that arrests haustorium invasion (Hegenauer et al. 2016, Hegenauer et al. 2020). The other resistance example occurs in certain hybrid cultivars of tomato and confers resistance to *C. campestris* through an enhanced lignification response that is coordinately regulated by four genes (Jhu et al. 2022). Both of these mechanisms provide clues to genes that could be used to confer *Cuscuta* resistance to susceptible crop species.

Investigations into parasite-inducible regulatory elements have received less attention. Nevertheless, insights into parasite-responsive elements may be gleaned from previous studies aimed at understanding host defense responses and how parasitic angiosperms compare to other classes of plant pathogens. For example, a tomato gene, *3-hydroxy-3-methylglutaryl CoA reductase* (*HMGR*) (Cramer et al. 1993), was characterized during host–parasite interactions using tobacco (*Nicotiana tabacum*) hosts engineered with the regulatory region of the defense-related version of this gene (*hmg2*) fused to the β-glucuronidase (GUS) reporter gene. When parasitized by *Orobanche aegyptiaca* (Syn. *Phelipanche aegyptiaca*), the GUS expression was localized to the region immediately around the site of parasite invasion (Westwood et al. 1998). Similarly, *Phelipanche aegyptiaca* parasitism induced the expression of a basic pathogenesis-related (PR) protein gene (Joel and Portnoy 1998) and genes related to phenylpropanoid metabolism (Griffitts et al. 2004). Notably, such genes are induced by other stimuli and are not specific to parasitism, but they indicate that host plants recognize parasitic plants as invaders and induce the expression of defense-related genes in response. If engineered resistance mechanisms are to be effective, they should be expressed specifically at the point of parasite haustorial attachment to avoid the metabolically wasteful and potentially harmful effects of inappropriately deployed defense responses.

Gene regulatory elements are well studied. In plants the main functional unit is the core promoter, situated about 35 bases in front of the transcription start site (TSS). This consists of the TATA box, flanked by the B recognition element that binds part of the transcription factor II (TFII) complex that in turn recruits RNA polymerase II for transcription, and the Y patch (pyrimidine-rich section) that stabilizes transcriptional machinery binding (Jores et al. 2021, Marand et al. 2023, Yasmeen et al. 2023). Further upstream is the proximal promoter region with *cis*-regulatory elements (CREs), and then the distal promoter region further upstream. The CREs influence gene expression by binding TFs that contribute to inducing or repressing gene expression. TFs specifically interact with transcription factor binding sites (TFBSs), which occur widely in gene regulatory regions. Given that TFBSs may range in length from 5 to 15 bases (Harbison et al. 2004), a typical promoter region of 1–2 Kb may contain over a hundred potential TFBSs. While TFs are the focus of the current work, it is noteworthy that transcription is also influenced by factors such as chromatin accessibility and DNA methylation status.

Studies of gene regulation have typically used full-length native promoters, but there is increasing interest in understanding specific functional gene regulatory elements that can be manipulated with greater ease and precision (Jores et al. 2021, Yasmeen et al. 2023, Borowsky and Bailey-Serres 2024). Most native gene regulatory regions have been honed by evolution to provide sophisticated gene regulation, but their expression patterns are not entirely specific and may function in multiple cell types and at multiple growth stages due to the complex interplay of factors involved in transcriptional regulation. A simplified approach to controlling gene expression is to identify specific TFBSs and place them in the context of a minimal promoter, which contains only the essential core elements to drive gene expression. Studies of such “minimal synthetic promoters” have revealed key aspects of promoter design, allowing for precise spatial and temporal control of transgene expression (Cominelli et al. 2011, Hou et al. 2012, Brückner et al. 2015, Ali and Kim 2019, Cai et al. 2020). These have been used to generate simple promoters with useful patterns of expression ranging from constitutive to tissue-specific or stress-inducible (Yasmeen et al. 2023).

In this study, we set out to identify gene regulatory elements that are specifically induced by *C. campestris* that could function to drive the expression of engineered defense responses. We began by identifying parasite-induced genes from an RNAseq study in *Arabidopsis thaliana*. The regulatory regions of two genes were analyzed to identify TFBSs, which were then recombined into a set of minimal synthetic inducible promoters (MSIPs). The MSIPs were fused to the GUS reporter gene, transformed into *A. thaliana*, and characterized for their response to parasitism. Here, we show that stress-induced TFBSs show the greatest specificity for parasitism and demonstrate the power of MSIPs for engineering the precise regulation of gene expression.

## Results

### Identification of functional elements in promoters

The first step in identifying parasite-inducible genes was an analysis of data from previous research that explored mobile gene transcripts in the *Arabidopsis*−*Cuscuta* interactions (Kim et al. 2014). Comparisons of expressed *A. thaliana* genes revealed two genes that were highly expressed in haustorial-invaded tissues while having low expression in adjacent stem tissues. Both genes were cytochrome P450 monooxygenase enzymes, CYP71A13 and *Phytoalexin Deficient 3* (*PAD3*, CYP71B15). These genes both function in the pathway leading to camalexin biosynthesis and were previously characterized as having low expression except when induced by pathogens (Schuhegger et al. 2006, Frerigmann et al. 2015). We analyzed the promoter sequences from these genes to identify common TFBSs. Each promoter contained numerous TFBSs, which we categorized according to their reported responses to stimuli, mostly falling into three broad groups: (i) stress, (ii) light, or (iii) anaerobic conditions (Supplementary Table S1). We then designed MSIPs that featured TFBS members of these groups by response category. Because single TFBSs are not as effective as clusters (Cai et al. 2020), three TFBSs were used either in combinations of different types or as repeats of the same type.

The basic MSIP design was based on previously reported synthetic promoters (Brückner et al. 2015, Cai et al. 2020). These promoters consist of 19 bps of random sequence at the 5′ end, followed by a second sequence of flexible length into which TFBSs may be inserted, followed by a consensus TATA box sequence (TATATAA) and a 43 bp minimal core ending with a TSS (Fig. 1a). We constructed five MSIPs (i.e. MSIP-01 to MSIP-05) that contained different combinations of TFBSs (Fig. 1b). TFBSs for stress, light, and anaerobic responses were arranged either by their response category (MSIP-01, -02, and -03) or in combinations to respond to multiple categories. Specifically, MSIP-04 contained all the TFBSs from MSIP-01 to -03, while MSIP-05 included all these plus an additional TFBS for the “vascular system,” which was not present in the native cytochrome P450 promoters but was added to explore the hypothesis that vascular-associated gene expression could be involved in response to the phloem-feeding *Cuscuta*. We also generated a negative control, MSIP-06, which lacked the TATA box.

**Figure 1:**
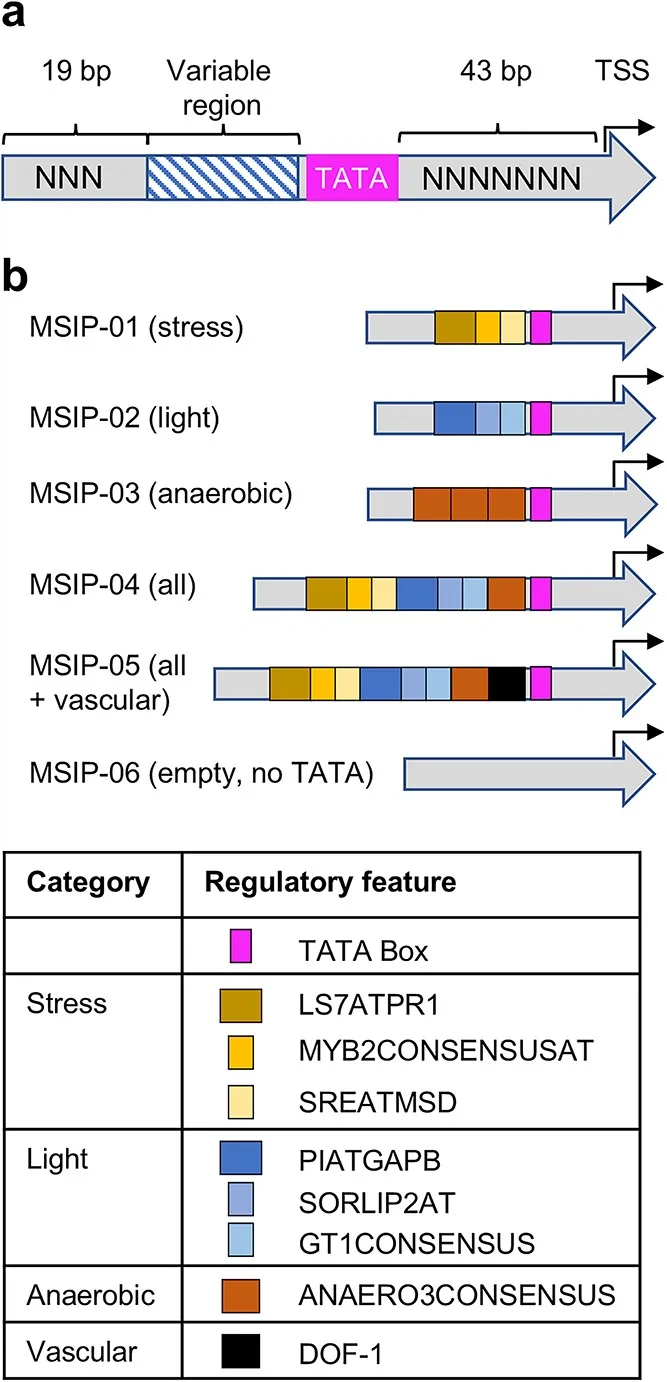
Design of MSIPs. (a) General design of MSIPs. TSS. (b) Specific designs for six MSIPs used in this study, with colored boxes indicating TFBSs and the TATA box.

### Induction of MSIPs by *C. campestris* parasitism in *A. thaliana*

The transcriptional activity of the MSIPs was evaluated in transgenic *A. thaliana* plants by assaying GUS activity at regions of *C. campestris* haustorial invasion and in other tissues. No GUS activity was detected in any tissues of the wild-type (WT) *A. thaliana* (Fig. 2a−c) or in a line with the promoter lacking the TATA box (MSIP-06, Fig. 2s−u) or one transformed with the promoterless plasmid (Supplementary Fig. S1). GUS expression was observed in the *A. thaliana* lines MSIP-01, MSIP-02, MSIP-03, and MSIP-04 (Fig. 2d, g, j, m). For each of these, GUS activity was induced in stems parasitized by *C. campestris* and was localized to the region of haustorial invasion. Non-parasitized stems showed no GUS activity (data not shown). The MSIP-05 construct did not show expression in response to the parasite or in leaves or reproductive tissues. The MSIP lines displayed variations in GUS expression patterns, ranging from weak to strong expression of the blue GUS product. MSIP-01, with stress-inducible TFBSs, had the strongest expression associated with parasitism (Fig. 2d). MSIP-02, -03, and -04 also showed parasite-specific expression, although it was generally fainter than in MSIP-01. Apart from the region of parasite connection, GUS staining revealed that non-parasitized leaves and siliques had low and seemingly random patches of expression. In leaves, some small patches of blue were observed, most commonly in MSIP-04 as shown in Fig. 2n. Notably, the lines that responded to *C. campestris* parasitism (MSIP-01, -02, -03, and -04) also showed expression in reproductive tissues. This was most pronounced at the junction of the silique and pedicel, and to a lesser extent, at the silique tip.

**Figure 2:**
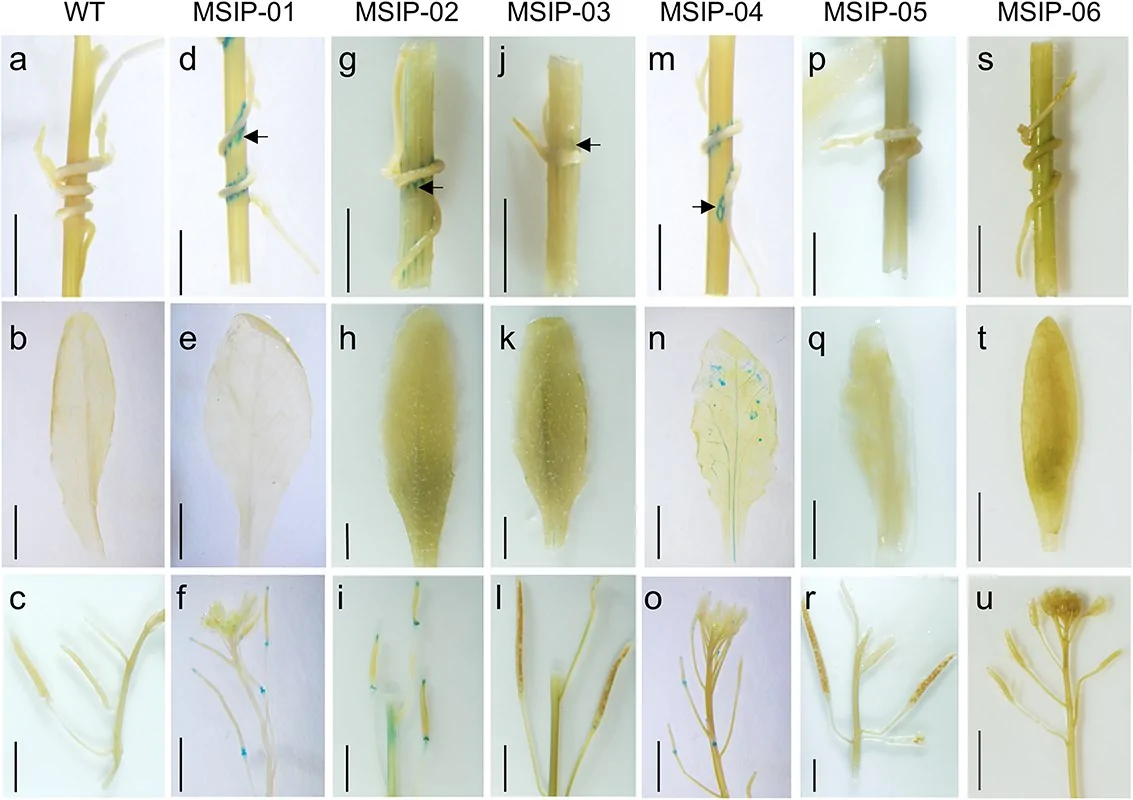
Patterns of expression of MSIP:GUS constructs in lines of *A. thaliana* parasitized by *C. campestris*. Top row (a, d, g, j, m, p, s): floral shoot stems with parasite connections. Middle row (b, e, h, k, n, q, t): leaves. Bottom row (c, f, i, l, o, r, u): inflorescences with seed pods. WT plant and MSIP line numbers are arranged in columns. Scale bars represent 0.5 cm. Arrows indicate blue associated with parasitism.

As a counterpoint to these novel promoter designs, we also analyzed *A. thaliana* plants expressing GUS under the control of the cauliflower mosaic virus 35S promoter (CaMV35S), which is widely used for driving constitutive gene expression (Amack and Antunes 2020). The 35S:GUS plants showed widespread expression of GUS in the leaves and floral tissues, but no blue color was observed at the points of haustorial attachment (Supplementary Fig. S1). Together, these patterns indicate that the MSIPs produce specific, reproducible patterns of expression.

### MSIP expression through early development

To further characterize the specificity of the MSIPs, we analyzed GUS expression in the transgenic lines at developmental stages ranging from seeds to young rosettes, all in the absence of *C. campestris* parasitism. GUS activity was not generally detected in imbibing or germinating seeds or in seedlings (Fig. 3). However, faint GUS expression was observed in the emerging radicle in MSIP-01 and MSIP-02 (Fig. 3a). GUS expression was observed at the shoot−root junction in MSIP-04 plants at the stage where cotyledons were fully open. In young plants with 5−8 rosette leaves, MSIP lines -02 and -04 showed GUS activity in distinct patterns (Fig. 3b). Blue staining was present in the central axis and specific leaves in MSIP-04, with a similar pattern but reduced intensity in MSIP-02. Faint expression in the stem was detected in MSIP-01 and MSIP-03. No GUS activity was detected in MSIP-05 or the WT *A. thaliana*.

**Figure 3:**
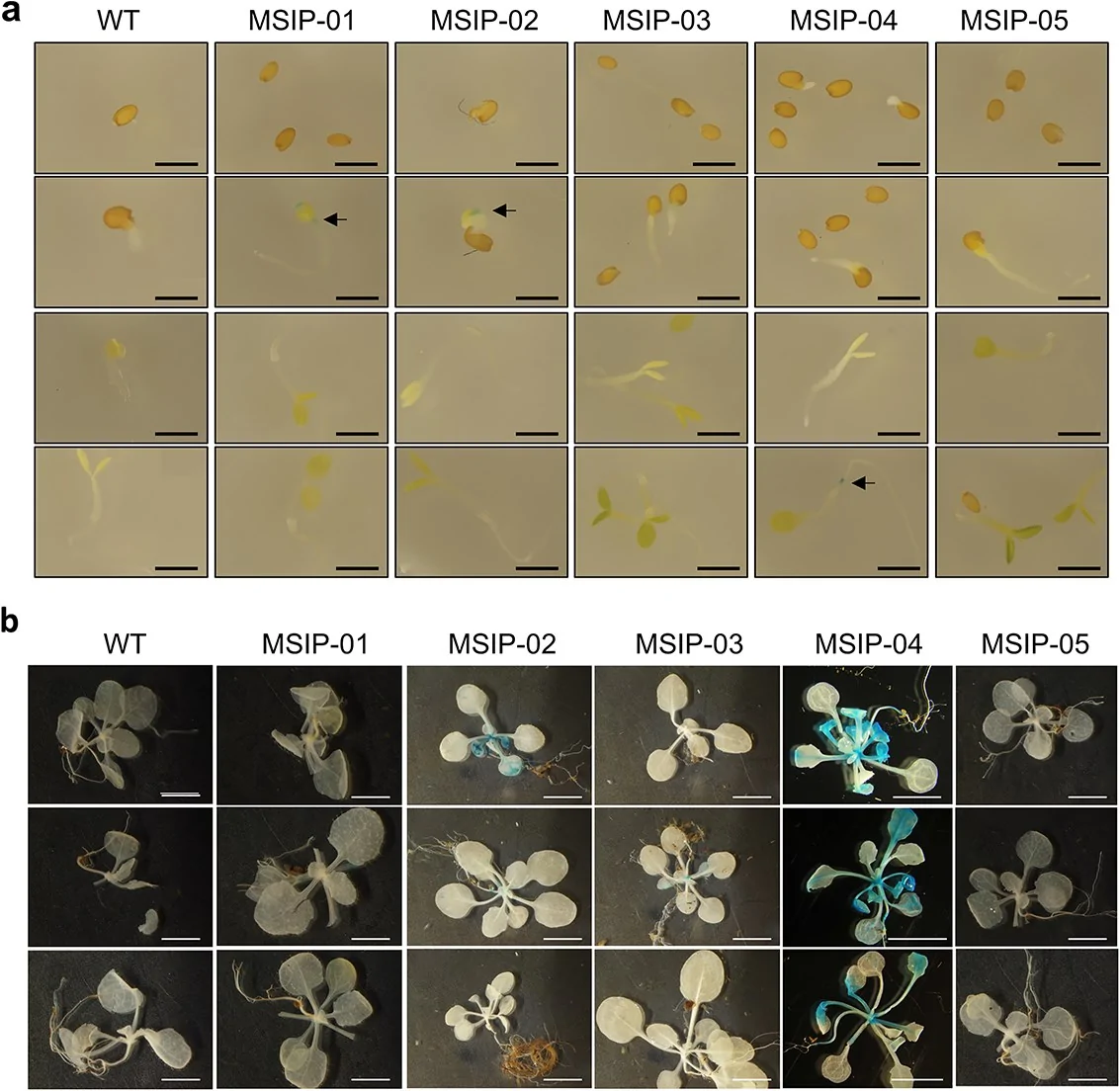
GUS staining of WT and MSIP:GUS lines of *A. thaliana* during vegetative developmental stages without stress. (a) Early development from imbibed seeds (top row) through radicle emergence and unfolded cotyledons (bottom row). Scale bar is 1 mm. (b) Seedlings with 5−8 rosette leaves. Three biological replicates are represented for each promoter construct. Scale bar is 0.5 cm.

### Detailed characterization of MSIP-01

The strong induction of GUS in the region of parasitism in MSIP-01, combined with relatively low expression in unaffected tissues, prompted a deeper investigation into this line. These results suggested a tightly controlled expression associated with parasitism and raised the potential utility for MSIP-01 as a *C. campestris-*driven gene regulator*.*

#### Time course expression during parasitism

To better understand the timing and cell specificity of the promoter response to parasitism, a time-course analysis was conducted. Vegetative cuttings of *C. campestris* were attached to floral stems of MSIP-1 *A. thaliana* and allowed to parasitize the host over the course of 12 days. Tissues were evaluated for GUS expression using the 4-methylumbelliferyl-β-D-glucuronide (MUG) fluorometric assay for quantification and the 5-Bromo-4-chloro-3-indolyl b-D-glucuronide, sodium salt (X-gluc) assay to visualize expression (Cervera 2004). The parasite–host associations were characterized by an examination of external surfaces and by cross-sections through the host stem at the point of *C. campestris* parasitism. The development of *Cuscuta* haustoria is divided into three stages: (i) adhesive phase (i.e. establishing of a connection with the host tissue); (ii) intrusive phase (invasion of haustoria into the host tissue); and (iii) conductive stage (transmission of nutrients through a functional haustorium) (Shimizu and Aoki 2019). The adhesive phase covers Days 0–2, during which the *C. campestris* stem coils around the host stem, but has not yet penetrated the host tissue. Visually, no GUS expression was detected either at the external surface or in cross-section until Day 4 (Fig. 4a), although in a previous study GUS activity was observed at Day 3 (data not shown). This agrees with the MUG assay, which indicated GUS expression as early as Days 1 and 2 (Fig. 4b). The intensity of GUS expression as determined by blue pigmentation increased through Day 7 and was sustained throughout the course of 12 days, with a slight decrease in color intensity in cross sections. The MUG assay captured a slightly different pattern of enzyme activity, including the earlier induction of gene expression as well as sustained activity that continued to increase at Day 12. These results are consistent with the MSIP-01 promoter responding to parasite intrusion as well as persisting during the conductive phase. During haustorial invasion the GUS signal initiates in the host cells around the haustorium, with expression in the epidermal and cortical cells. The expression intensifies and appears to spread to neighboring cells, but it remains concentrated in the region of haustorial invasion. Expression does not appear to be associated with the vascular tissue in the *A. thaliana* stem. Most of the host pith cells were lost during sectioning, but there was no indication of GUS expression in those that remained.

**Figure 4:**
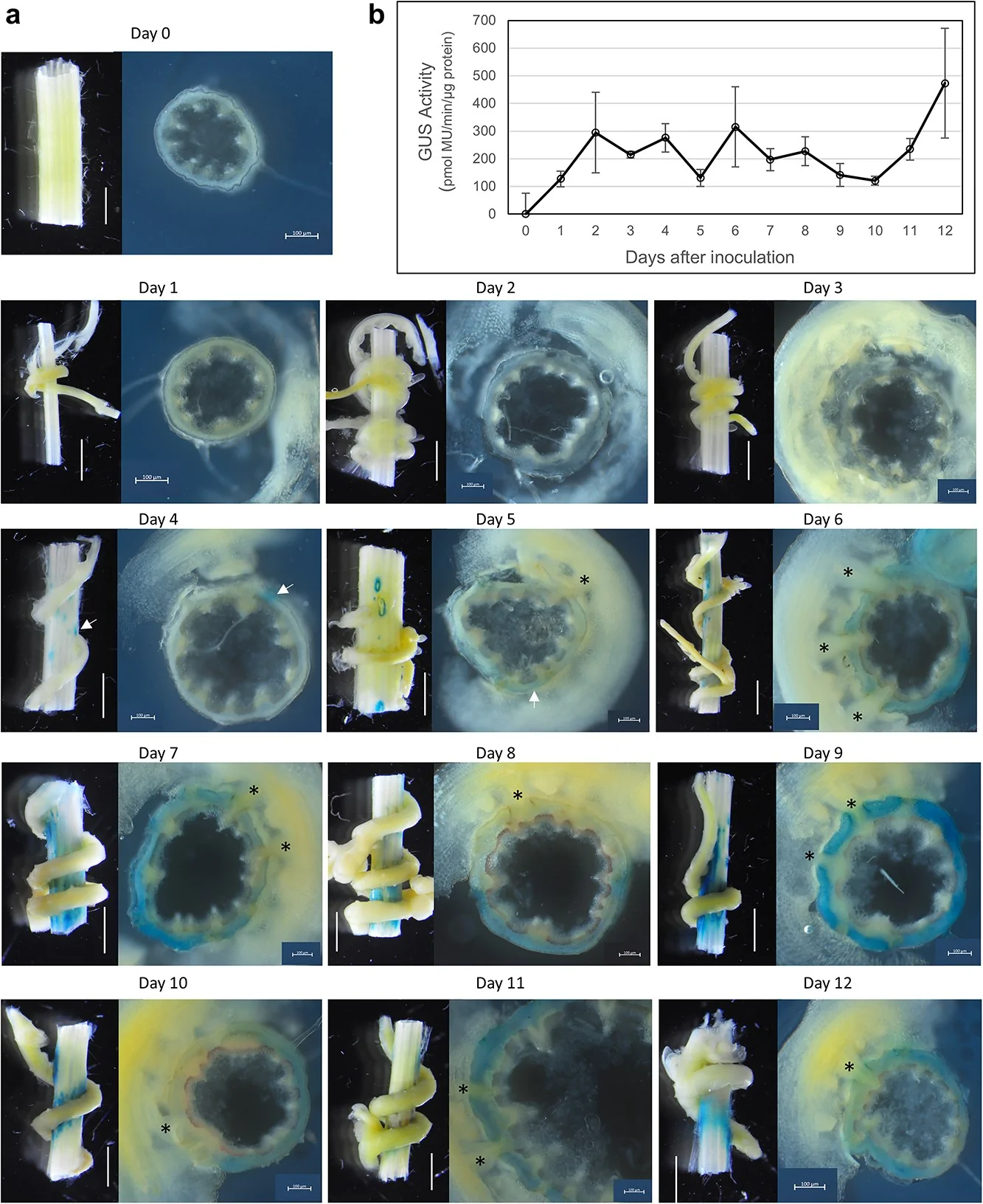
Time course of MSIP-01 expression over 12 days in response to *C. campestris* parasitism. (a) Localization of GUS expression associated with parasitism as revealed in surface view and in host cross section at each time point. Parasite is not attached at Day 0, but is present at Day 1. Haustoria have formed by Day 3, with penetration of host at Day 4. Haustoria examples are marked by ^*^. For surface photos scale bars are 2 mm. (b) MUG assay quantifying GUS activity at each day. Data are means of three replicates with bars representing standard error.

#### Response to wounding and crushing

Previous studies established that parasitism induces genes that may be involved in localized defense responses to stress and pathogen attack (Joel and Portnoy 1998, Westwood et al. 1998, Vieira Dos Santos et al. 2003, Griffitts et al. 2004). To understand the triggers of MSIP-01 expression, we assayed the promoter response to mechanical wounding. Two types of wounding were evaluated, cutting with a sharp blade, or crushing of the plant stems or leaves. Expression of GUS in the MSIP-01:GUS transgenic plants occurred for both types of wounds (Fig. 5a, Supplementary Fig. S2a). The response was more pronounced in the stems than in the leaves, with crushing eliciting a stronger response than cutting, perhaps due to more cells being affected in the crushing treatment. In general, the GUS expression was restricted to the area of injury and did not induce a systemic response.

**Figure 5:**
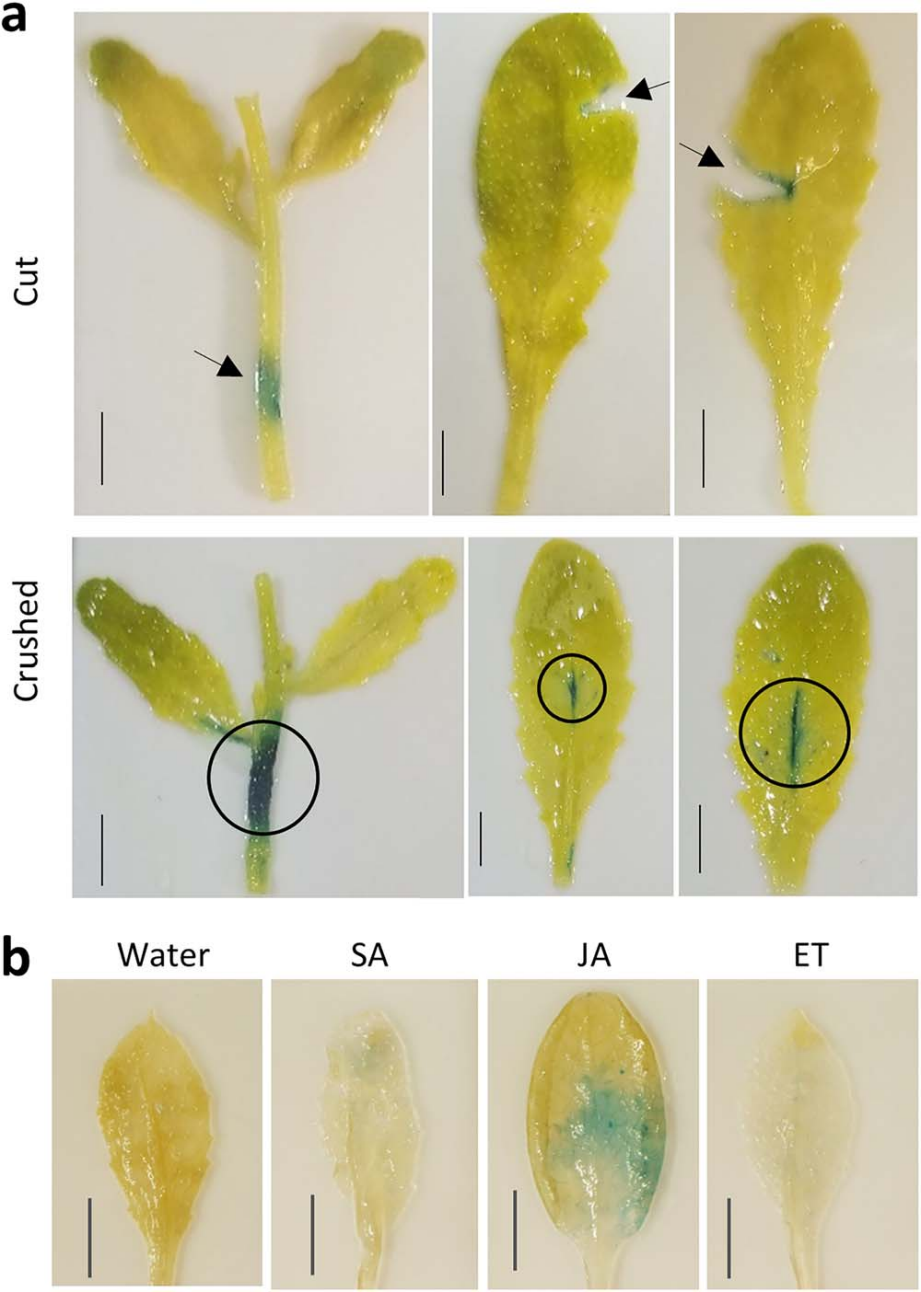
MSIP-01:GUS response to injury and chemical triggers. (a) Physical injury from cutting with a razor blade (top) or crushing (bottom). Arrows and circles indicate points of wounding. (b) Exposure to defense-inducing chemicals: water control, SA, JA, and ET. Scale bars are 5 mm.

#### Response to salicylic acid, methyl jasmonate, and ethylene

Next, we evaluated the MSIP-01 response to the phytohormones jasmonic acid (JA), salicylic acid (SA), and ethylene (ET), which play major roles in regulating defense gene expression (Li et al. 2019). SA is a critical signaling molecule for triggering PR protein transcription and resistance against pathogen infection. Both the ET and JA signaling pathways are required for the activation of plant defense against necrotrophic pathogens. We tested leaves of the MSIP-01-GUS transgenic plants in the presence of methyl jasmonate, SA and ET. GUS activity was clearly present in leaves treated with methyl jasmonate, but only at lower levels in leaves treated with SA and ET (Fig. 5b, Supplementary Fig. S2b). The CYP71A13 and *PAD3* genes that inspired the MSIP designs are characterized by their response to AgNO_3_ treatment. Similar treatment of leaves with 5 mM AgNO_3_ did not induce GUS expression in MSIP-01 (data not shown), suggesting that the response to AgNO_3_ is mediated through other TFBSs in the native promoter that were not among the elements in MSIP-01.

### MSIP responses to the root parasite *P. aegyptiaca*

*P. aegyptiaca* (Egyptian broomrape) is another parasitic plant species that represents a distinct lineage of parasitism from *Cuscuta. P. aegyptiaca* attacks the roots of a host plant rather than the shoots parasitized by *Cuscuta*. Both parasites form haustoria to attach to their hosts, but the exact mechanisms by which the haustoria integrate with host tissues are poorly understood, and few studies have sought to compare haustorial function between different types of parasitic plants. One previous study showed that an arabinogalactan protein (attAGP)-promoter from *S. lycopersicum* is activated by both *C. reflexa* parasitism and *P. aegyptiaca* (Rehker et al. 2012)*.* Considering this report, and that both types of parasites invade by physically disrupting host tissue, we asked how our set of MSIPs would respond to *P. aegyptiaca* parasitism of their roots.

The transgenic *A. thaliana* expressing GUS driven by the MSIPs were grown in a semi-hydroponic system and inoculated with seeds of *P. aegyptiaca*. After infection by *P. aegyptiaca,* the roots were stained for GUS expression. The WT control plant did not show any staining at the site of *Phelipanche* attachment, while most of the MSIP lines produced GUS expression at the point of parasite attachment and in patches away from the point of attachment (Fig. 6). Roots of the MSIP-01 lines again showed parasite-induced expression with little GUS staining distant from the point of the haustorial connection. Lines MSIP-01, -02, -03, and -04 had clear GUS expression at the points of *Phelipanche* attachment, though staining was observed extending further from the attachment site and in areas not parasitized by *P. aegyptiaca*. No GUS activity was observed for MSIP-05 in roots with or without the parasite.

**Figure 6:**
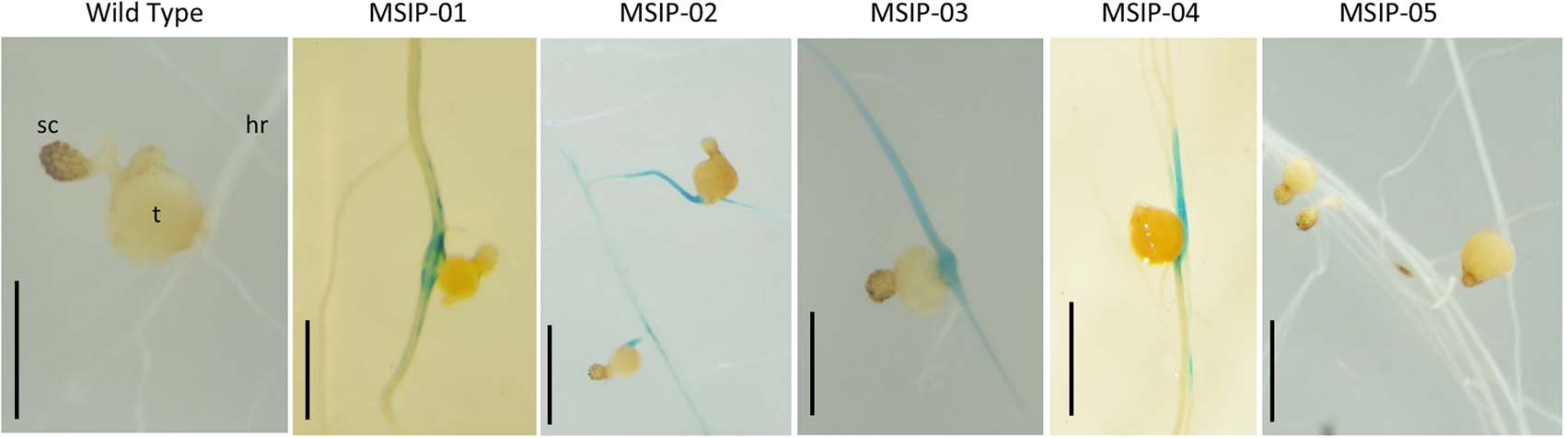
Patterns of expression of MSIPs in response to the root parasite *P. aegyptiaca*. Structures labeled in the WT image are host root (hr), parasite seed coat (sc), and parasite tubercle (t). Scale bars are 1 mm.

## Discussion

The predictable and precise control of gene expression is central to the effective use of biotechnology for crop improvement. In this study, we aimed to develop an MSIP for gene expression with a high degree of specificity in response to *C. campestris* parasitism. Our MSIP designs were based on the *cis*-regulatory regions of two *A. thaliana* genes, CYP71A13 and CYP71B15, that showed strong *C. campestris*-induced expression (Kim et al. 2014). The TFBSs within the CREs from these genes were identified and incorporated into a set of MSIPs, grouping the TFBSs by their reported responsiveness to factors such as stress, light, anaerobic conditions, vascular tissue, or combinations of these (Fig. 1). With one exception, all the resulting MSIPs were responsive to *C. campestris* parasitism as evidenced by GUS staining at the point of haustorial attachment to the host stem (Fig. 2). The induction of GUS expression associated with stress, light, and anaerobic conditions indicates the broad impact of *C. campestris* on host transcriptional networks. Interestingly, combining all the TFBSs into the same promoter, MSIP-04, did not confer a substantial increase in parasite responsiveness, although TFBSs do not always function in an additive manner (Cominelli et al. 2011). However, the incorporation of multiple different TFBSs drives stronger expression than repeated copies of the same TFBS (Cai et al. 2020), perhaps explaining the relatively low expression of GUS in MSIP-03 that contained three copies of the same anaerobic-responsive TFBS (Fig. 2). The exception to these patterns of parasite inducibility was MSIP-05, which contained a combination of all the TFBSs (as in MSIP-04) plus a vascular specific TFBS, yet no expression was observed in this line. The vascular-related TFBS selected was predicted to bind a transcription factor named DNA binding with One Finger (DOF-1), which is expressed in vascular tissue and involved in vascular cell development and function in *A. thaliana* (Ranjan and Dey 2012, Le Hir and Bellini 2013, Ramachandran et al. 2020). The contrast of a lack of any GUS signal in MSIP-05 compared to the relatively widespread expression in MSIP-04 suggests that the DOF-1 TFBS in this context may recruit a transcriptional repressor. DOF transcription factors are reported to include both positive and negative regulators of gene expression (Hidalgo and Lohmann 2026).

Regarding specificity in their response to parasitism, all MSIPs from -01 to -04 showed GUS expression in addition to that induced at the points of haustorial attachment. All of these had strong expression at the extreme ends of siliques, most prominently at abscission zone at the junction with the pedicel (Fig. 2f, i, l, o). Some also exhibited GUS activity in mature leaves (e.g. Fig. 2n) or at various stages of seedling development (Fig. 3). Nevertheless, the promoter with the most specific expression associated with *C. campestris* attachment was MSIP-01. It combined a strong response to the parasite with low expression in other tissues and across a range of developmental stages (Figs. 2 and 3). In contrast, MSIPs containing the light-responsive TFBSs (MSIP- 02 and -04) exhibited various levels of GUS expression in the leaf and petiole tissues. MSIP-03 showed low levels of GUS expression in general, including in response to the parasite, suggesting that the anaerobic elements or the repetition of the TFBS promoted only weak gene expression. The strong expression of MSIP-01 in the pedicel abscission zone is intriguing and potentially problematic for the use of this promoter in engineered parasite resistance strategies. Gene expression is highly active in the pedicel and includes ET responsive genes (Wang et al. 2013), whose regulatory networks may interact with the TFBSs incorporated in MSIP-01. This raises the possibility that using MSIP-01 to drive the expression of a highly detrimental gene could affect silique development.

MSIP-01 was further characterized as a *C. campestris* inducible promoter. The exact onset of MSIP-01 driven GUS expression in response to parasitism was within 1 day of parasite coiling as determined by the MUG assay (Fig. 4b), and at 3–4 days according to the appearance of blue color in the tissues (Fig. 4a). The MUG assay is more sensitive than GUS staining (Cervera 2004), but such a rapid response is surprising considering that haustoria do not develop until 3 days after initial coiling. Nevertheless, host gene expression changes in response to parasitism by *Phelipanche ramosa* and *Orobanche cumana* can occur within hours of parasite exposure (Vieira Dos Santos et al. 2003, Letousey et al. 2007). The induction of a host gene in response to the coiling of the *C. campestris* suggests a similarly high degree of sensitivity by the host. At the other end of the time course, the MUG assay indicated high levels of activity on Day 12, suggesting continued expression of the GUS gene. This was consistent with the continued presence of the blue color in the region of parasite invasion. The MUG assay showed a high degree of variation between biological replicates, reflecting our observations that, while the MSIP-01 promoter was highly consistent in the timing and pattern of expression, the magnitude of GUS expression varied between samples.

The invasion by haustoria triggered GUS staining primarily in the cortex layer of the *A. thaliana* stem (Fig. 4). The molecular interactions between *Cuscuta* spp. and their hosts are complex, potentially involving multiple layers of signaling. In addition to the physical stress of wounding as the parasite loosens walls and forces aside host cells (Vaughn 2003, Johnsen et al. 2015), the parasite and host plants undergo extensive interplant trafficking of systemic signals, secondary metabolites, mRNAs, small RNAs, and proteins (Haupt et al. 2001, Kim et al. 2014, Smith et al. 2016, Hettenhausen et al. 2017, Shahid et al. 2018, Liu et al. 2020), each of which has the potential to impact gene expression in the recipient organism. The host response to this attack is likely to be similarly complex. Our results point to a combination of three stress-response-related TFBSs as being especially specific to *C. campestris* parasitism. LS7ATPR1 is found in promoters associated with the ET response (Ahmadizadeh et al. 2020) and in the SA-mediated NPR1 induction of PR-1 (Johnson et al. 2003). MYB2CONSENSUSAT is associated with the promoters of drought-responsive genes (Yamamoto et al. 2007), and SREATMSD is involved in sugar signaling (Tatematsu et al. 2005). Of these, the LS7ATPR1 element appears to be the most related to pathogen defense, but in MSIP-01 it does not confer a significant response to either SA or ET (Fig. 5b). Rather, exposure to JA or physical wounding by cutting or crushing was more effective in generating TFs to promote GUS expression. Removing these TFBSs from the context of a native promoter and recombining them in the MSIPs may affect their responses, although the overall effect agrees with reports of the root parasite *Phelipanche* primarily inducing JA-related defenses (Westwood et al. 1998, Vieira Dos Santos et al. 2003).

Our studies also point to similarities between *C. campestris* and *P. aegyptiaca* in terms of MSIP responses (Figs. 1 and 6). The finding that both parasites trigger similar sets of TFs is remarkable considering that these two species represent unrelated lineages of parasitism, attack different plant organs, and have different haustorial structures. But at the level of cell–cell interactions, both parasites must achieve the same objective of remodeling host cell walls, integrating with host physiology, and evading host defenses. MSIP-01 again showed the most parasite-associated GUS expression in response to the parasitism of roots by *P. aegyptiaca*. This is consistent with the report of an attAGP gene in *S. lycopersicum* being induced in response to parasitism by *C. reflexa* (Albert et al. 2006) as well as by *P. aegyptiaca* (Rehker et al. 2012). Subsequent transcriptomic studies using these parasites (e.g. Runyon et al. 2010, Torres-Vera et al. 2016) include other examples of similarities in the host response, but none have yet examined the gene regulatory regions in detail. These results suggest that there may be a high degree of parallel evolution in how parasitic plant haustoria interact with their hosts.

MSIPs are potentially useful in engineering host resistance because they simplify transgene expression and may have broad application to different host species. First, synthetic promoters have advantages in regulating gene expression in constitutive, inducible, or tissue-specific patterns (Ali and Kim 2019). For regulating defense responses against pathogens, inducible promoters are preferred because they avoid the metabolic cost and misdirected defenses that are associated with constitutive gene over-expression (van Hulten et al. 2006). Another advantage is that a small synthetic promoter does not contain excess TFBSs that may be targeted by TFs that repress or otherwise modify gene expression (Borowsky and Bailey-Serres 2024). Thus, MSIPs should not be easily suppressed by parasite strategies to manipulate host defense signaling networks (Clarke et al. 2020). Furthermore, TFBSs tend to be evolutionarily conserved (Lambert et al. 2019), so they may be expected to retain their regulatory properties across species, for example functioning similarly in *A. thaliana* and tobacco (Jores et al. 2021). Finally, the small size of MSIPs contributes to cheaper and more efficient cloning protocols.

In summary, we have studied several MSIPs in response to parasitism and identified one, MSIP-01, as being highly correlated with the type of damage inflicted by the parasitism of both *C. campestris* and *P. aegyptiaca*. This promoter should be evaluated for its ability to drive the expression of genes hypothesized to have a function in host defense against parasitism. Currently, few single genes are known to produce an effective defense against parasitic plants, but MSIP-01 could be a component of transgenic approaches to evaluate candidate resistance genes.

## Materials and Methods

### Plant material and growth conditions

*A. thaliana* (Col-0) was used as the host plant for this study. *C. campestris* seeds were from a line propagated in the Westwood laboratory and previously characterized for parasite–host interactions, though erroneously reported as *Cuscuta pentagona* (Kim et al. 2014, Shahid et al. 2018). Nurseries of the parasite were established on beets (*Beta vulgaris*, Detroit Dark Red), and vegetative cuttings of *C. campestris* shoot tips ~6 cm long were used for experiments by taping the parasite shoots to the floral stem of an *A. thaliana* host plant. The parasite shoots were allowed to coil around the host stems and form haustorial connections. Non-parasitized control plants were grown under the same conditions as the parasitized plants but were not exposed to *C. campestris*. Six days after parasite placement on the hosts, tissues from plants that had established *C. campestris* with at least two coils around healthy host stems and clear parasite growth were harvested. The experiment on root-parasite induction of promoters used *P. aegyptiaca* (lot 32142 courtesy of D. Joel, A.R.O. Newe Ya’ar, Israel) grown on *A. thaliana* plants in a semi-hydroponic polyethylene (PE) bag system as previously described (Westwood et al. 1998).

### Identification of candidate TFBSs

Data from a previous study by Kim et al. (2014) were used to identify *A. thaliana* genes having expression triggered by *C. campestris* parasitism. Genes were sorted to identify those that were highly expressed in the haustorial−host interface region while having low expression in adjacent host stem tissue. The two *A. thaliana* genes selected were CYP71A13 (AT2G30770) and CYP71B15 (AT3G26830). Promoter CRE analysis was performed using PlantPAN 2.0 (http://PlantPAN2.itps.ncku.edu.tw) to identify co-occurring TFBSs in the promoters of the query genes (CYP71A13 and CYP71B15). Gene identifiers corresponding to the target genes were submitted to the Gene Group Search module, and the promoter regions were analyzed using the default PlantPAN settings for the selected plant species. The resulting TFBS co-occurrence output was exported and manually curated. TFBSs were selected based on their frequency within the analyzed promoters and their reported association with defense signaling, stress responses, anaerobic regulation, and pathogen-/parasite-induced gene regulation in plants. Functional annotations and descriptions of the TFBSs were compiled using PlantPAN annotations and supporting literature references. The identified TFBSs were subsequently classified into four functional categories: stress-related, light-responsive, anaerobic-responsive, and other regulatory elements. Among these categories, stress-related TFBSs represented the largest group (14 TFBSs), followed by light-responsive elements (9 TFBSs), whereas anaerobic-responsive elements were less represented (3 TFBSs). Additional TFBSs classified as other regulatory elements accounted for 9 TFBSs. Due to their greater representation and potential biological relevance to parasite-responsive promoter activity, stress-related, light-responsive, and anaerobic-responsive TFBSs were prioritized for further interpretation and discussion. The light-responsive TFBSs were analyzed separately from the general stress-related category because the identified motifs were primarily associated with photoregulation, circadian rhythm, phytochrome signaling, etiolation, and shade avoidance responses rather than general abiotic stress pathways such as drought or mechanical stress. The number of repeats for each TFBS within the promoter regions of the query genes was additionally counted and tabulated separately. The co-occurrence of TFBSs was determined and candidate TFBSs were categorized as being related to stress, light, or anaerobic conditions (Supplementary Tables S1 and S3). The top candidates (with the highest occurrence) were selected for use in the MSIP.

Five constructs were initially designed according to their predicted response to: stress (MSIP-01), light (MSIP-02), anaerobic (MSIP-03), combined stress, light, and anaerobic (MSIP-04), and the combined TFBSs used in MSIP-04 plus a vascular TFBS (MSIP-05). We evaluated a panel of control constructs that included one designated MinSyn-000 by Cai et al. (2020) as a negative control promoter, but we found that even this contained parasite-responsive TFBSs. Ultimately, we generated a negative control consisting of the truncated promoter lacking the TATA box (MSIP-06) and the promoterless pEarleyGate303-GUSPlus plasmid. As a constitutive expression control, we also analyzed *A. thaliana* plants expressing GUS under the CaMV35S, which is widely used to drive strong, ubiquitous gene expression (Amack and Antunes 2020).

### Construction of MSIPs

pEarleyGate303-GUSPlus plasmid was used as the backbone for all constructs. This vector was built by cloning the GUS gene in pEarleyGate303 (Earley et al. 2006). To construct the plasmids harboring the MSIPs, we cloned the promoter fragments upstream of the GUS gene. The MSIP sequences were split into two segments that were included in the forward and reverse primers accordingly (Supplementary Table S2). The primers were phosphorylated using polynucleotide kinase (New England Biolabs) and the plasmid pEarleyGate303-GUSPlus was PCR amplified. The obtained PCR product was ligated with the instant sticky-end ligase (New England Biolabs) and was transformed into *Escherichia coli* DB3.1. Plasmids were then transformed into *Agrobacterium tumefaciens* (GV3101).

### Production of stable transformants

Transformation of *A. thaliana* was performed by the floral dip method (Zhang et al. 2006). Liquid cultures of *A. tumefaciens* were grown from single colonies in a growth medium supplemented with 50 μg/mL rifampicin, 25 μg/mL gentamycin, and 50 μg/mL kanamycin at 28°C. *A. tumefaciens* cells were collected by centrifugation and resuspended in a 5% (wt/vol) sucrose solution and Silwet L-77 at a concentration of 0.02% (vol/vol). *Arabidopsis* plants were inverted and dipped in the *Agrobacterium* cell suspension for 10 s with gentle agitation. The dipped plants were covered with plastic film and maintained for 24 h. Seeds were collected from mature siliques and surface sterilized. Selection of transgenic plants was performed on Modified Murashige and Skoog media (MMS) media supplemented with 10 mg L^−1^ Basta. *E. coli* colonies were picked for each construct and were checked for the presence of the exact promoter and at the expected size. Sequencing of the transgene inserts was repeated for the *A. thaliana* transgenic plants to confirm the transformations. In addition to these lines, non-transformed WT *A. thaliana* served as a negative control. For each gene construct, four independent transgenic events were generated, selected, and characterized. Exceptions were MSIP-04 (three lines) and MSIP-06 (five lines). Expression patterns were similar within each construct.

### Histochemical GUS staining

For the detection of GUS activity, tissues were incubated overnight at 37°C in GUS staining solution: 2 mM X-gluc (Biosynth Carbosynth), 0.1% (v/v) Triton X-100 (Sigma-Aldrich), 10 mM EDTA (Thermo Fisher Scientific), 0.3 M mannitol (Sigma-Aldrich), and 50 mM sodium phosphate buffer, pH 7. Samples were cleared by soaking in 70% ethanol for 24 h and then examined using an Olympus SZX16 stereomicroscope.

### Time course expression during parasitism

*C. campestris* attachments were made onto the MSIP-01 transgenic lines and were monitored for 12 days. Day 0 of the time-course was defined as the day that a vegetative cutting of *C. campestris* was taped to the *A. thaliana* inflorescence stem, and this served as a baseline for comparison to subsequent time points (recognizing that a full parallel series of non-parasitized/mock-taped stems could further the parasite response from any wounding associated with inoculation). Day 1 was defined as the day where *Cuscuta* had successfully coiled around the host stem, within 24 h of placement. Parasite cuttings were applied every day such that all tissues could be harvested and processed on the same day. For the quantitative assay of GUS activity, three biological replicates were collected, each comprising 2–4 host stem pieces (0.5–0.75 cm long) containing *C. campestris* connections and representing 1–2 host plants. The fluorometric assay of GUS was conducted as described by Cervera (2004) with minor modifications. Freshly harvested tissues were immediately placed in 400 μL of cold extraction buffer (50 mM sodium phosphate buffer at pH 7.0, 10 mM Na_2_EDTA at pH 8.0, 0.1% Triton X-100, and 10 mM β-mercapto-ethanol) in a 1.5 mL centrifuge tube and ground to lyse cell. Extracts were centrifuged at 4°C and 13 000 g for 10 min to remove debris, and were assayed by mixing 150 μL extract with 150 μL MUG substrate (2 mM). Reactions were incubated at 37°C, and 100 μL aliquots were removed at 10, 20, and 30 min and mixed with 100 μL stop solution (0.2 M Na_2_CO_3_, pH 9.5). Fluorescence of the product MU (4-methylumbelliferone) was measured in a plate reader (Synergy HTX, Agilent), set at 365 nm excitation and 455 nm emission. The protein content of each extract was determined by the Bradford method (Bradford 1976) using a commercially prepared reagent (BioRad Protein Assay Dye Reagent Concentrate). Enzyme activity was expressed in pmol MU/min/μg total protein.

For spatial characterization, sections of *A. thaliana* stem with attached *C. campestris* were harvested and stained in X-gluc solution and destained as described above. The external appearance of the parasite–host interactions were documented using a Zeiss Stemi SV11 stereomicroscope. Next, samples were embedded in 5% agarose and were sectioned with a VT1200 S fully automated vibrating blade microtome (Leica) at 400 μm thickness and 0.5 mm/sec speed with 0.8 mm amplitude. Sectioned tissues were then visualized using a Zeiss AX10 compound microscope.

### Wounding and crushing treatments

MSIP-01 stems and leaves were subjected to wounding with a razor blade without detaching the stem or leaf from the plant. Crushing was performed by applying fingertip pressure to the point of cell damage. Stems and leaves were collected and stained with GUS staining solution as described above.

### SA, methyl jasmonate, and ET treatments

Leaves, stems, and flowers of MSIP-01 plants were incubated for 1 h in solutions of 1 mM SA, 1 mM methyl jasmonate, or 5 mM ET. Tissues were then transferred to water and incubated for 23 h. Samples were stained with GUS staining solution as described above.

### Assay with the root parasite *P. aegyptiaca*

*A. thaliana* MSIP and WT plants were grown in pots in a growth chamber until the 4–5 leaves stage. Then, the plants were transplanted into a semi-hydroponic PE bag system (Westwood et al. 1998), to enable the observation of different stages of development of *Phelipanche*. Plants were grown for a week in the PE bags to adapt to the growth conditions and then were inoculated with *P. aegyptiaca* seeds that had been surface cleaned, conditioned for 1 week, and stimulated to germinate with 1 ppm GR-24. Plants were maintained by the addition of ¼ X Hoagland solution (Hoagland and Arnon 1950) to the bags as needed. Conditioned *Phelipanche* seeds usually start germinating 4–5 days after exposure to GR-24, and roots with attached parasite tubercles were observed 8–10 days after inoculation. Root segments of ~5 cm in length with *Phelipanche* attachments were separated from the main plant for GUS staining and observed under the microscope as described above.

## Supplementary Material

Supplementary_File_pcag101

## Data Availability

All data supporting the findings of this study are included in the main text and supplementary materials.

## References

[ref1] Ahmadizadeh, M., Chen, J.-T., Hasanzadeh, S., Ahmar, S. and Heidari, P. (2020) Insights into the genes involved in the ethylene biosynthesis pathway in *Arabidopsis thaliana* and *Oryza sativa*. J. Genet. Eng. Biotechnol. 18: 62. 10.1186/s43141-020-00083-1 PMC7572930 33074438

[ref2] Albert, M., Belastegui-Macadam, X. and Kaldenhoff, R. (2006) An attack of the plant parasite *Cuscuta reflexa* induces the expression of attAGP, an attachment protein of the host tomato. Plant J. 48: 548–556. 10.1111/j.1365-313X.2006.02897.x 17076801

[ref3] Albert, M., Axtell, M.J. and Timko, M.P. (2020) Mechanisms of resistance and virulence in parasitic plant–host interactions. Plant Physiol. 185: 1282–1291. 10.1093/plphys/kiaa064 PMC8133583 33793887

[ref4] Ali, S. and Kim, W.C. (2019) A fruitful decade using synthetic promoters in the improvement of transgenic plants. Front. Plant Sci. 10: 14. 10.3389/fpls.2019.01433 PMC6838210 31737027

[ref5] Aly, R., Matzrafi, M. and Bari, V.K. (2021) Using biotechnological approaches to develop crop resistance to root parasitic weeds. Planta 253: 97. 10.1007/s00425-021-03616-1 33844068

[ref6] Amack, S.C. and Antunes, M.S. (2020) CaMV35S promoter – a plant biology and biotechnology workhorse in the era of synthetic biology. Cur. Plant Biol. 24: 100179. 10.1016/j.cpb.2020.100179

[ref7] Borowsky, A. and Bailey-Serres, J. (2024) Rewiring gene circuitry for plant improvement. Nat. Genet. 56: 1574–1582. 10.1038/s41588-024-01806-7 39075207

[ref8] Bradford, M. (1976) A rapid and sensitive method for the quantitation of microgram quantities of protein utilizing the principle of protein-dye binding. Anal. Biochem. 72: 248–254. 10.1016/0003-2697(76)90527-3 942051

[ref9] Brückner, K., Schäfer, P., Weber, E., Grützner, R., Marillonnet, S. and Tissier, A. (2015) A library of synthetic transcription activator-like effector-activated promoters for coordinated orthogonal gene expression in plants. Plant J. 82: 707–716. 10.1111/tpj.12843 PMC4691316 25846505

[ref10] Cai, Y.M., Kallam, K., Tidd, H., Gendarini, G., Salzman, A. and Patron, N.J. (2020) Rational design of minimal synthetic promoters for plants. Nucleic Acids Res. 48: 11845–11856. 10.1093/nar/gkaa682 PMC7708054 32856047

[ref11] Cervera, M. (2004) Histochemical and fluorometric assays for uidA (GUS) gene detection. In Transgenic Plants: Methods and Protocols. Edited by Peña, L. pp. 203–213. Humana Press, Totowa, NJ, 10.1385/1-59259-827-7:203 15310923

[ref12] Clarke, C.R., Park, S.Y., Tuosto, R., Jia, X., Yoder, A., Van Mullekom, J., et al. (2020) Multiple immunity-related genes control susceptibility of *Arabidopsis thaliana* to the parasitic weed *Phelipanche aegyptiaca*. PeerJ 8: e9268. 10.7717/peerj.9268 PMC7289146 32551199

[ref13] Cominelli, E., Galbiati, M., Albertini, A., Fornara, F., Conti, L., Coupland, G., et al. (2011) DOF-binding sites additively contribute to guard cell-specificity of AtMYB60promoter. BMC Plant Biol. 11: 162. 10.1186/1471-2229-11-162 PMC3248575 22088138

[ref14] Costea, M. and Tardif, F.J. (2006) The biology of Canadian weeds. 133. *Cuscuta campestris* Yuncker, *C. Gronovii* Willd. Ex Schult., *C. Umbrosa* Beyr. Ex hook., *C. Epithymum* (L.) L. and *C. Epilinum* Weihe. Can. J. Plant Sci. 86: 293–316. 10.4141/P04-077

[ref15] Cramer, C.L., Weissenborn, D.L., Cottingham, C.K., Denbow, C.J., Eisenback, J.D., Radin, D.N., et al. (1993) Regulation of defense-related gene expression during plant-pathogen interactions. J. Nematol. 25: 507–518. https://pmc.ncbi.nlm.nih.gov/articles/PMC2619434/PMC2619434 19279804

[ref16] Cuccurullo, A., Nicolia, A. and Cardi, T. (2022) Resistance against broomrapes (*Orobanche* and *Phelipanche* spp.) in vegetables: a comprehensive view on classical and innovative breeding efforts. Euphytica 218: 82. 10.1007/s10681-022-03035-7

[ref17] Earley, K.W., Haag, J.R., Pontes, O., Opper, K., Juehne, T., Song, K., et al. (2006) Gateway-compatible vectors for plant functional genomics and proteomics. Plant J. 45: 616–629. 10.1111/j.1365-313X.2005.02617.x 16441352

[ref18] Eizenberg, H., Hershenhorn, J., Ephrath, J.H. and Kanampiu, F. (2013) Chemical control. In Parasitic Orobanchaceae: Parasitic mechanisms and control strategies. Edited by Joel, D.M., Gressel, J. and Musselman, L.J. pp. 415–432. Springer, Berlin, 10.1007/978-3-642-38146-1_23

[ref19] Fernández-Aparicio, M., Delavault, P. and Timko, M.P. (2020) Management of infection by parasitic weeds: a review. Plants 9: 1184. 10.3390/plants9091184 PMC7570238 32932904

[ref20] Frerigmann, H., Glawischnig, E. and Gigolashvili, T. (2015) The role of MYB34, MYB51 and MYB122 in the regulation of camalexin biosynthesis in *Arabidopsis thaliana*. Front. Plant Sci. 6: 654. 10.3389/fpls.2015.00654 PMC4548095 26379682

[ref21] Furuhashi, T., Furuhashi, K. and Weckwerth, W. (2011) The parasitic mechanism of the holostemparasitic plant *Cuscuta*. J. Plant Interact. 6: 207–219. 10.1080/17429145.2010.541945

[ref22] Griffitts, A.A., Cramer, C.L. and Westwood, J.H. (2004) Host gene expression in response to Egyptian broomrape (*Orobanche aegyptiaca*). Weed Sci. 52: 697–703. 10.1614/WS-03-155R

[ref23] Harbison, C.T., Gordon, D.B., Lee, T.I., Rinaldi, N.J., Macisaac, K.D., Danford, T.W., et al. (2004) Transcriptional regulatory code of a eukaryotic genome. Nature 431: 99–104. 10.1038/nature02800 PMC3006441 15343339

[ref24] Haupt, S., Oparka, K.J., Sauer, N. and Neumann, S. (2001) Macromolecular trafficking between *Nicotiana tabacum* and the holoparasite *Cuscuta reflexa*. J. Exp. Bot. 52: 173–177. 10.1093/jexbot/52.354.173 11181727

[ref25] Hegenauer, V., Fürst, U., Kaiser, B., Smoker, M., Zipfel, C., Felix, G., et al. (2016) Detection of the plant parasite *Cuscuta reflexa* by a tomato cell surface receptor. Science 353: 478–481. 10.1126/science.aaf3919 27471302

[ref26] Hegenauer, V., Slaby, P., Körner, M., Bruckmüller, J.-A., Burggraf, R., Albert, I., et al. (2020) The tomato receptor CuRe1 senses a cell wall protein to identify Cuscuta as a pathogen. Nat. Commun. 11: 5299. 10.1038/s41467-020-19147-4 PMC7576778 33082345

[ref27] Hettenhausen, C., Li, J., Zhuang, H., Sun, H., Xu, Y., Qi, J., et al. (2017) Stem parasitic plant *Cuscuta australis* (dodder) transfers herbivory-induced signals among plants. Proc. Natl. Acad. Sci. U. S. A. 114: E6703–E6709. 10.1073/pnas.1704536114 PMC5559024 28739895

[ref28] Hidalgo, I. and Lohmann, J.U. (2026) The role of DOF transcription factors in generating specificity during plant development. J. Exp. Bot. erag012. 10.1093/jxb/erag012 41549457

[ref29] Hoagland, D.R. and Arnon, D.I. (1950) The water-culture method for growing plants without soil. Calif. Agric. Exp. Sta. Circ. 347: 1–32.

[ref30] Hou, L., Chen, L., Wang, J., Xu, D., Dai, L., Zhang, H., et al. (2012) Construction of stress responsive synthetic promoters and analysis of their activity in transgenic *Arabidopsis thaliana*. Plant Mol. Biol. Rep. 30: 1496–1506. 10.1007/s11105-012-0464-0

[ref31] Jhu, M.-Y., Farhi, M., Wang, L., Philbrook, R.N., Belcher, M.S., Nakayama, H., et al. (2022) Heinz-resistant tomato cultivars exhibit a lignin-based resistance to field dodder (*Cuscuta campestris*) parasitism. Plant Physiol. 189: 129–151. 10.1093/plphys/kiac024 PMC9070836 35099559

[ref32] Joel, D.M. and Portnoy, V.H. (1998) The angiospermous root parasite *Orobanche* L. (Orobanchaceae) induces expression of a pathogenesis related (PR) gene in susceptible tobacco roots. Ann. Bot. 81: 779–781. 10.1006/anbo.1998.0629

[ref33] Johnsen, H.R., Striberny, B., Olsen, S., Vidal-Melgosa, S., Fangel, J.U., Willats, W.G.T., et al. (2015) Cell wall composition profiling of parasitic giant dodder (*Cuscuta reflexa*) and its hosts: a priori differences and induced changes. New Phytol..: n/a–n/a 207: 805–816. 10.1111/nph.13378 25808919

[ref34] Johnson, C., Boden, E. and Arias, J. (2003) Salicylic acid and NPR1 induce the recruitment of trans-activating TGA factors to a defense gene promoter in *Arabidopsis*. Plant Cell 15: 1846–1858. 10.1105/tpc.012211 PMC167174 12897257

[ref35] Jores, T., Tonnies, J., Wrightsman, T., Buckler, E., Cuperus, J., Fields, S., et al. (2021) Synthetic promoter designs enabled by a comprehensive analysis of plant core promoters. Nat. Plants 7: 842–+. 10.1038/s41477-021-00932-y PMC10246763 34083762

[ref36] Kim, G., LeBlanc, M.L., Wafula, E.K., dePamphilis, C.W. and Westwood, J.H. (2014) Genomic-scale exchange of mRNA between a parasitic plant and its hosts. Science 345: 808–811. 10.1126/science.1253122 25124438

[ref37] Lambert, S.A., Yang, A.W.H., Sasse, A., Cowley, G., Albu, M., Caddick, M.X., et al. (2019) Similarity regression predicts evolution of transcription factor sequence specificity. Nat. Genet. 51: 981–989. 10.1038/s41588-019-0411-1 31133749

[ref38] Le Hir, R. and Bellini, C. (2013) The plant-specific Dof transcription factors family: new players involved in vascular system development and functioning in *Arabidopsis*. Front. Plant Sci. 4: 2013. 10.3389/fpls.2013.00164 PMC3665933 23755058

[ref39] Letousey, P., De Zélicourt, A., Vieira Dos Santos, C., Thoiron, S., Monteau, F., Simier, P., et al. (2007) Molecular analysis of resistance mechanisms to *Orobanche cumana* in sunflower. Plant Pathol. 56: 536–546. 10.1111/j.1365-3059.2007.01575.x

[ref40] Li, N., Han, X., Feng, D., Yuan, D. and Huang, L.-J. (2019) Signaling crosstalk between salicylic acid and ethylene/jasmonate in plant defense: do we understand what they are whispering? Int. J. Mol. Sci. 20: 671. 10.3390/ijms20030671 PMC6387439 30720746

[ref41] Liu, N., Shen, G., Xu, Y., Liu, H., Zhang, J., Li, S., et al. (2020) Extensive inter-plant protein transfer between *Cuscuta* parasites and their host plants. Molec. Plant 13: 573–585. 10.1016/j.molp.2019.12.002 31812691

[ref42] Marand, A.P., Eveland, A.L., Kaufmann, K. and Springer, N.M. (2023) Cis-regulatory elements in plant development, adaptation, and evolution. Ann. Rev. Plant Biol. 74: 111–137. 10.1146/annurev-arplant-070122-030236 PMC9881396 36608347

[ref43] Masanga, J., Mwangi, B.N., Kibet, W., Sagero, P., Wamalwa, M., Oduor, R., et al. (2021) Physiological and ecological warnings that dodders pose an exigent threat to farmlands in eastern Africa. Plant Physiol. 185: 1457–1467. 10.1093/plphys/kiab034 PMC8355486 33661304

[ref44] Olsen, S., Striberny, B., Hollmann, J., Schwacke, R., Popper, Z. and Krause, K. (2015) Getting ready for host invasion: elevated expression and action of xyloglucan endotransglucosylases/hydrolases in developing haustoria of the holoparasitic angiosperm *Cuscuta*. J. Exp. Bot. 67: 695–708. 10.1093/jxb/erv482 PMC4737069 26561437

[ref45] Parker, C. (2012) Parasitic weeds: a world challenge. Weed Sci. 60: 269–276. 10.1614/WS-D-11-00068.1

[ref46] Ramachandran, V., Tobimatsu, Y., Masaomi, Y., Sano, R., Umezawa, T., Demura, T., et al. (2020) Plant-specific Dof transcription factors VASCULAR-RELATED DOF1 and VASCULAR-RELATED DOF2 regulate vascular cell differentiation and lignin biosynthesis in *Arabidopsis*. Plant Mol. Biol. 104: 263–281. 10.1007/s11103-020-01040-9 32740898

[ref47] Ranjan, R. and Dey, N. (2012) Development of vascular tssue and sress inducible hybrid–synthetic promoters through Dof-1 motifs tearrangement. Cell Biochem. Biophys. 63: 235–245. 10.1007/s12013-012-9359-9 22610660

[ref48] Rehker, J., Lachnit, M. and Kaldenhoff, R. (2012) Molecular convergence of the parasitic plant species *Cuscuta reflexa* and *Phelipanche aegyptiaca*. Planta 236: 557–566. 10.1007/s00425-012-1626-x 22460777

[ref49] Runyon, J.B., Mescher, M.C., Felton, G.W. and De Moraes, C.M. (2010) Parasitism by *Cuscuta pentagona* sequentially induces JA and SA defence pathways in tomato. Plant Cell Environ. 33: 290–303. 10.1111/j.1365-3040.2009.02082.x 19930126

[ref50] Scholes, J.D. and Press, M.C. (2008) *Striga* infestation of cereal crops - an unsolved problem in resource limited agriculture. Curr. Opin. Plant Biol. 11: 180–186. 10.1016/j.pbi.2008.02.004 18337158

[ref51] Schuhegger, R., Nafisi, M., Mansourova, M., Petersen, B.L., Olsen, C.E., Svatoš, A., et al. (2006) CYP71B15 (PAD3) Catalyzes the final step in camalexin biosynthesis. Plant Physiol. 141: 1248–1254. 10.1104/pp.106.082024 PMC1533948 16766671

[ref52] Shahid, S., Kim, G., Johnson, N.R., Wafula, E., Wang, F., Coruh, C., et al. (2018) MicroRNAs from the parasitic plant *Cuscuta campestris* target host messenger RNAs. Nature 553: 82–85. 10.1038/nature25027 29300014

[ref53] Shimizu, K. and Aoki, K. (2019) Development of parasitic organs of a stem holoparasitic plant in genus *Cuscuta*. Front. Plant Sci. 10: 11. 10.3389/fpls.2019.01435 PMC6861301 31781146

[ref54] Smith, J.D., Woldemariam, M.G., Mescher, M.C., Jander, G. and De Moraes, C.M. (2016) Glucosinolates from host plants influence growth of the parasitic plant *Cuscuta gronovii* and its susceptibility to aphid feeding. Plant Physiol. 172: 181–197. 10.1104/pp.16.00613 PMC5074629 27482077

[ref55] Spallek, T., Mutuku, M. and Shirasu, K. (2013) The genus *striga*: a witch profile. Molec. Plant Pathol. 14: 861–869. 10.1111/mpp.12058 PMC6638688 23841683

[ref56] Tatematsu, K., Ward, S., Leyser, O., Kamiya, Y. and Nambara, E. (2005) Identification of cis-elements that regulate gene expression during initiation of axillary bud outgrowth in *Arabidopsis*. Plant Physiol. 138: 757–766. 10.1104/pp.104.057984 PMC1150394 15908603

[ref57] Torres-Vera, R., García, J.M., Pozo, M.J. and López-Ráez, J.A. (2016) Expression of molecular markers associated to defense signaling pathways and strigolactone biosynthesis during the early interaction tomato-*Phelipanche ramosa*. Physiol. Mol. Plant Pathol. 94: 100–107. 10.1016/j.pmpp.2016.05.007

[ref58] van Hulten, M., Pelser, M., van Loon, L.C., Pieterse, C.M.J. and Ton, J. (2006) Costs and benefits of priming for defense in *Arabidopsis*. Proc. Natl. Acad. Sci. U. S. A. 103: 5602–5607. 10.1073/pnas.0510213103 PMC1459400 16565218

[ref59] Vaughn, K.C. (2003) Dodder hyphae invade the host: a structural and immunocytochemical characterization. Protoplasma 220: 189–200. 10.1007/s00709-002-0038-3 12664283

[ref60] Vieira Dos Santos, C., Letousey, P., Delavault, P. and Thalouarn, P. (2003) Defense gene expression analysis of *Arabidopsis thaliana* parasitized by *Orobanche ramosa*. Phytopathology 93: 451–457. 10.1094/PHYTO.2003.93.4.451 18944360

[ref61] Wang, X., Liu, D., Li, A., Sun, X., Zhang, R., Wu, L., et al. (2013) Transcriptome analysis of tomato flower pedicel tissues reveals abscission zone-specific modulation of key meristem activity genes. PLoS One 8: e55238. 10.1371/journal.pone.0055238 PMC3563536 23390523

[ref62] Westwood, J.H., Yu, X., Foy, C.L. and Cramer, C.L. (1998) Expression of a defense-related 3-hydroxy-3-methylglutaryl CoA reductase gene in response to parasitization by Orobanche spp. MPMI 11: 530–536. 10.1094/MPMI.1998.11.6.530 9612951

[ref63] Yamamoto, Y.Y., Ichida, H., Matsui, M., Obokata, J., Sakurai, T., Satou, M., et al. (2007) Identification of plant promoter constituents by analysis of local distribution of short sequences. BMC Genomics 8: 67. 10.1186/1471-2164-8-67 PMC1832190 17346352

[ref64] Yasmeen, E., Wang, J., Riaz, M., Zhang, L. and Zuo, K. (2023) Designing artificial synthetic promoters for accurate, smart, and versatile gene expression in plants. Plant Commun. 4: 100558. 10.1016/j.xplc.2023.100558 PMC10363483 36760129

[ref65] Zhang, X., Henriques, R., Lin, S.-S., Niu, Q.-W. and Chua, N.-H. (2006) *Agrobacterium*-mediated transformation of *Arabidopsis thaliana* using the floral dip method. Nature Protoc. 1: 641–646. 10.1038/nprot.2006.97 17406292

