# Supplementary material for: Minimal synthetic inducible promoters for host response to parasitic plants
Source: Plant Cell Physiol. 2026 Jul 20;67(10):1747–59. doi: 10.1093/pcp/pcag101 (PMC13714602; doi:10.1093/pcp/pcag101)
Supplement: Supplementary file 1 [file supplementary_file_pcag101.zip › Supplementary material_05-14-26.docx]

**Minimum Synthetic Inducible Promoters for Host Response to Parasites**

Imen Tanniche^1^, Hope Gruszewski^1^, Sukhmanpreet Kaur^2^, and James H. Westwood^1^

^1^School of Plant and Environmental Sciences, Virginia Tech

^2^Department of Biological Sciences, Virginia Tech

Contents: 2 Tables and 2 Figures.

Table S1. Analysis of TFBSs from *Cuscuta*-inducible promoters. Co-occurrence of TFBS in promoters of P450 and PAD3 genes based on PlantPAN 4.0 analysis (<https://plantpan.itps.ncku.edu.tw/plantpan4/index.html>). Classification of TFBSs into stress-related system (highlighted in orange), related to anaerobic genes (highlighted in green) and related light response (highlighted in blue). The number of times the TFBS was present in each promoter is indicated.

**Table S2.** Primers Sequences. Sequences comprising the MSIP regions are in Blue. The binding regions to the pEarleyGate303-GUSPlus plasmid backbone are shown in orange for forward and green for reverse primers. Uni: Universal reverse primers: MSIP_Uni-R1 was used for MSIP_neg to MSIP_03 amplifications and MSIP_Uni-R2 was used for MSIP_04 and MSIP_05 amplifications. Seq: Primers used to sequence promoters for construct verification.

| **Primer Name** | **Primer sequence** |
| --- | --- |
| MSIP_neg-F | TCCCCGCATAGCTGAACATCTATATAAGGTTTTGCTATTCATTGAAAGCAGTAGTGACTGATTTGTATATAAGTCTAGAATGGTAGATCTG |
| MSIP_01-F | ACGTCATAGACAACGGTTATCCAGCAAGACCTCTATATAAGGTTTTGCTATTCATTGAAAGCAGTAGTGACTGATTTGTATATA AGTCTAGAATGGTAGATCTG |
| MSIP_02-F | GTGATCACGGGCCGGAAAAAGCAAGACCTCTATATAAGGTTTTGCTATTCATTGAAAGCAGTAGTGACTGATTTGTATATA AGTCTAGAATGGTAGATCTG |
| MSIP_03-F | TCATCACTCATCACTCATCACAGCAAGACCTCTATATAAGGTTTTGCTATTCATTGAAAGCAGTAGTGACTGATTTGTATATA AGTCTAGAATGGTAGATCTG |
| MSIP_04-F | CCGTGATCACGGGCCGGAAAATCATCACAGCAAGACCTCTATATAAGGTTTTGCTATTCATTGAAAGCAGTAGTGACTGATTTGTATATA AGTCTAGAATGGTAGATCTG |
| MSIP_05-F | CCGTGATCACGGGCCGGAAAATCATCACAAAGAGCAAGACCTCTATATAAGGTTTTGCTATTCATTGAAAGCAGTAGTGACTGATTTGTATATA AGTCTAGAATGGTAGATCTG |
| MSIP_Uni-R1 | CCTCACTAAAACGACACGCCTGTTCCACCACTTTGTAC |
| MSIP_Uni-R2 | ATAACCGTTGTCTATGACGTCCTCACTAAAACGACACGC CTGTTCCACCACTTTGTAC |
| MSIP_06 -F | TCCCCGCATAGCTGAACATCTATATAAGGTTTTGCTATTCATTGAAAGCAGTAGTGACTGATTTGTATATA AGTCTAGAATGGTAGATCTG |
| MSIP_06 -R | CCTCGTATTCTGCGAGGGC CTGTTCCACCACTTTGTAC |
| MSIP-Seq-F | AGGTCGACCATAGTGACTGG |
| MSIP-Seq-R | CTCGGTGTTGATCGGGTAC |

**Figure S1.** Expression of additional constructs containing the GUS gene in *Arabidopsis*. (a,c,e) Promoterless GUS, (b,d,f) 35S:GUS. (a-b) *C. campestris* attachment region on *Arabidopsis* stem, (c-d) *Arabidopsis* leaf, and (e-f) inflorescence. Scale bar represents 0.5 cm.

**Figure S2.** MSIP-01:GUS response to injury and chemical triggers, with replicates. (a) Physical injury from cutting with a razor blade or crushing. Arrows and circles indicate points of wounding. Ruler divisions are 1 mm. (b) Exposure to defense-inducing chemicals: water control, salicylic acid, jasmonic acid, and ethylene. Scale bars are 5 mm.
